# Analysis of the Spatial Variability of Soil Texture in a Tropical Highland: The Case of the Jema Watershed, Northwestern Highlands of Ethiopia

**DOI:** 10.3390/ijerph15091903

**Published:** 2018-09-01

**Authors:** Mintesinot Taye, Belay Simane, Yihenew G. Selsssie, Benjamin Zaitchik, Shimelis Setegn

**Affiliations:** 1 Institute of Disaster Risk and Food Security Studies, Bahir Dar University, Bahir Dar 5501, Ethiopia; 2 College of Development Studies, Addis Ababa University, Addis Ababa 11079, Ethiopia; simaneb@yahoo.com; 3 College of Agriculture and Environmental Sciences, Bahir Dar University, Bahir Dar 5501, Ethiopia; yihenewgs@gmail.com; 4 Department of Earth and Planetary Sciences, Johns Hopkins University, Baltimore, MD 21218, USA; zaitchik@jhu.edu; 5 Environmental and Occupational Health, Florida International University, 11200 S.W. 8th St., Miami, FL 33199, USA; ssetegn@gmail.com

**Keywords:** soil texture, agro-climatic zones, spatial distribution, farmlands, Ethiopian highlands

## Abstract

This study sought to analyze the degree of spatial association of soil texture with agro-climatic zones and slope classes on the farmlands of the Jema watershed, in the Northwestern Highlands of Ethiopia. The agro-climatic zones (elevation zones) determine the micro-climate and biota of the study area. Thirty six soil composite samples for texture (the proportion of clay, silt and sand) analysis from four agro-climatic (elevation) zones and seven slope classes were collected. One-Way-ANOVA was employed to compute the mean variability of texture among the identified terrain classes, and linear regression was used to analyze the degree of association between texture and the terrain attributes. The measured values of sand, silt and clay in the watershed ranged from 11.4 to 43.4, 6.0 to 34.8, and 21.8 to 77.8, respectively. The One-Way-ANOVA indicated a significant (*p* < 0.05) soil texture variation in both slope and agro-climatic zone classes. Heavy clay, clay and clay loam were identified as the major texture classes in the lower, middle and upper parts of the watershed, respectively. The regression analysis showed that texture was more influenced by the difference in the elevation values than in slope values in the watershed. The standardized beta coefficients of slope and elevation for clay particles were 0.499 and 0.767, respectively. For sand, the regression coefficients for slope and agro-climatic zone were 0.485 and 0.812, respectively. This implies that an interactive effect of micro-climate and biota governed by elevation influenced the spatial distribution of soil texture more than slope.

## 1. Background 

Information on soil characteristics is a basic component of agricultural development programs and environmental regulation [1,2]. Information about soil is essential for improving the productivity and sustainability of farmlands [3,4]. However, the only horizontal soil information available in Ethiopia and other developing countries was the FAO’s 1974 world soil map done at a very small scale of 1:1,000,000 [5]. Recently, Berhanu et al. [6] organized, analyzed, and mapped a larger scale national soil map (1:250,000) by collecting information from FAO, MoWR and WBSP. The scale of the map was not large enough to characterize the soil texture and associated features of a smaller watershed like the study site. Lack of soil information is one of the constraints in the analysis of farmers’ livelihood vulnerability to climate variability [7,8] and land degradation [9,10,11,12] in Ethiopia. In the Lake Tana Sub-Basin, where the watershed is situated, sediment yield was roughly estimated to be above 30 tons/ha for the major watersheds [13]. However, this was an aggregated mean value computed for the larger study area where distinct watersheds are situated.

As stated in the work of Landon [14], available soil water content is greatly influenced by soil texture. Together with pore space, bulk density and organic matter, soil texture influences available water holding capacity, nutrient retention capacity, soil erodibility, and soil and water conservation planning [6]. Due to its larger pores, sandy soils have higher infiltration and less water holding capacity. The fine pore size of clayey soils is one of the major reasons for low water infiltration rate, high water holding capacity and less available water to plants. Keeping other factors constant, clayey soils do not drain more water which permits longer growing periods to plants [15]. Rugged terrain could cause an abrupt change in soil texture [5]. Tromp-van Meerveld et al. [16] and [17] also stated that soil texture varied spatially with terrain attributes.

Soil survey is an accurate but expensive task for developing countries [17]. Soil texture analysis with the help of terrain attributes is a cost effective strategy for conducting further pedotransfer analysis [15]. For predicting the pattern of soil properties over a landscape, catenary approach has been used as an analytical framework. In catenary soil development, there is a sequence of soils down a slope, created by the balance of processes such as precipitation, infiltration and runoff [18].

Elevation and slope were found to be among the main terrain attributes correlating with the spatial distribution of the soil groups [19]. However, the relationships between terrain attributes (elevation and slope) were not established yet [5,19]. Because of variation in complexity of terrain, parent material and microclimate, the task of defining the spatial distribution of soil properties including texture in farmlands located in various slope and elevation classes was not straightforward [12,20]. In the larger region of the study area, the Northwestern highlands of Ethiopia, there is lack of information on soil properties including soil texture to be used for land use management at watershed level [21]. Areas with higher elevation presented higher values of clay content [22]. On the other hand, Dessalegn et al. [23] showed that the loose aggregate nature of the upslope soils has enhanced easy movement of the fine soil particles downslope along with the rain water movement. Up till now there is no study that shows the degree of influence of agro-ecological zone (elevation class) and slope classes on the spatial distribution of soil texture [2]. It is important to note that elevation determines the spatial distribution of micro-climate, biota and farming practices in Ethiopian [4,24]. Soil parent materials and the factors mentioned above govern the degree of weathering and runoff [16]. Therefore, the objective of this study was to test the degree of spatial association of soil texture with agro-climatic zones (elevation) and slope classes on the farmlands of the Jema watershed in the Northwestern Highlands of Ethiopia.

## 2. Materials and Methods

### 2.1. Jema Watershed

**Figure 1:**
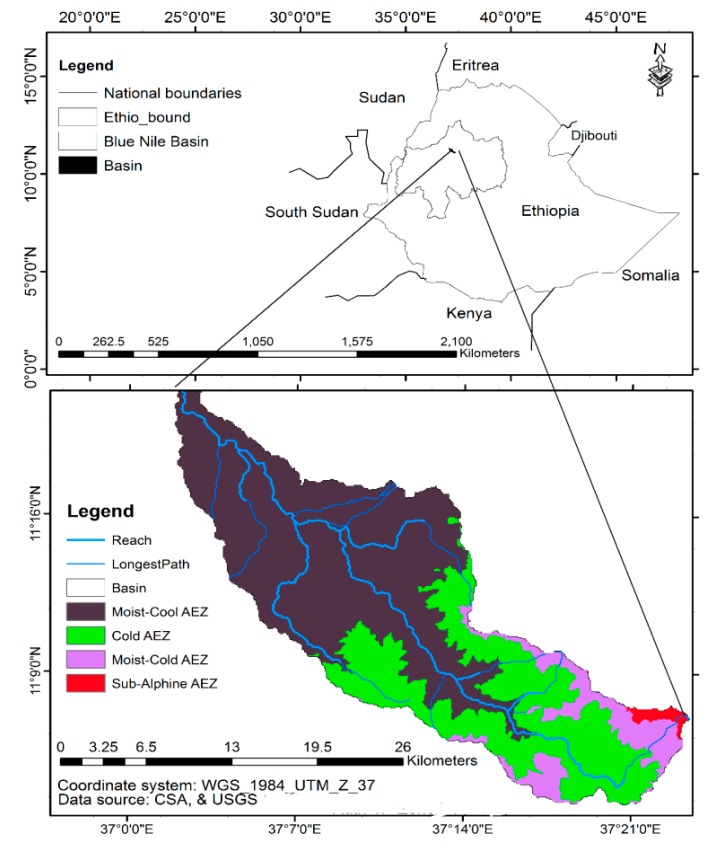
Location map of the Jema watershed.

**Figure 2:**
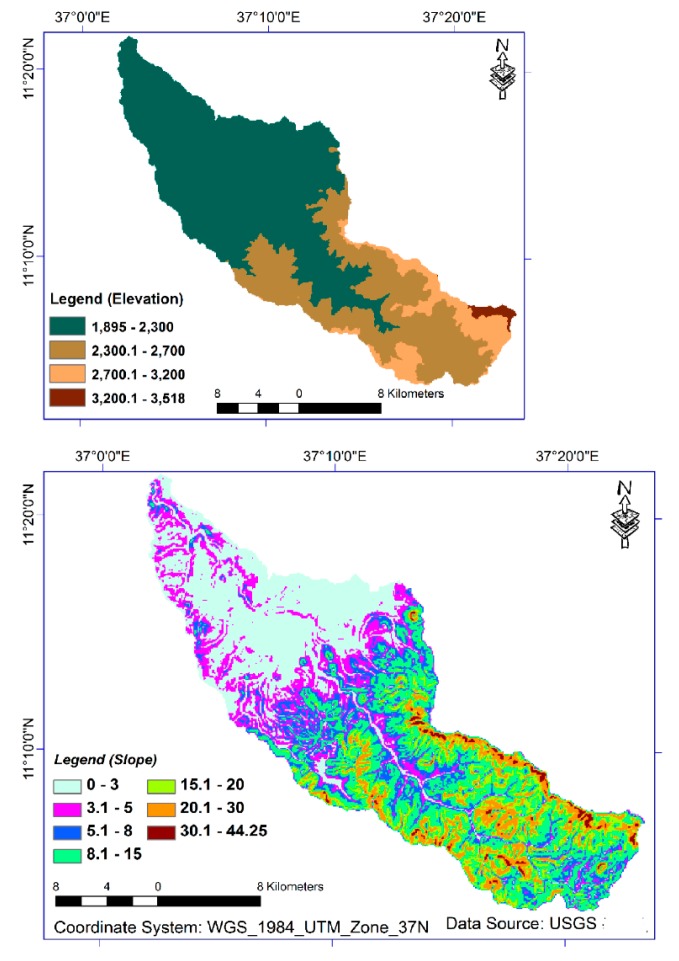
Elevation (agro-ecological zone) and slope class map of the Jema watershed.

The Jema watershed is situated in the Northwestern highlands of Ethiopia, the Upper Blue Nile River Basin in Lake Tana Sub-Basin (Figure 1). It has an area of 483 km^2^. Volcanic rocks of Late-Tertiary to Quaternary age dominate the geology of the watershed [25]. The site is characterized by flat, low-lying terrain consisting of plains, narrow stretches and rolling to hilly landscape at higher elevations. As per computed from DEM, with 30 meters resolution, the slope ranges from 0.2% to 44% and the elevation ranges from 1890 to 3525 meter above sea level (Figure 2). Based on Hurni’s [24] local agro-climatic classification, the watershed consists of moist-cool (woyina-dega), cold (dega), moist-cold (kefitegna-dega), sub-alpine (wurch) agro-climatic zones (Figure 2). Eighty five percent of the total precipitation occurs in the months of June, July and August with an annual average rainfall of 1500 mm. The daily mean maximum and minimum temperature are 27 °C and 9 °C, respectively [26]. The major soil orders are Alisols, Nitisols and Vertisols [27]. The features of the agro-climatic zones of the watershed are thought to be alike to the local agro-ecological zone features of Ethiopia (Table 1). The livelihoods of people in the watershed depend on food crop production and livestock rearing.

### 2.2. Data Collection

The soil sample sites and points were selected using a stratified and proportionate sampling techniques. The sample strata were prepared to consider the existing elevation (agro-climatic zones) and slope classes of farm lands of the Jema Watershed. Based on the FAO’guidelines [15], seven slope strata and four elevation sample strata were identified [24]. Samples were not collected on the steep slope (30–44.25%) due to the limited farmland presence. This terrain class map (raster layer) was generated from the DEM 30 meter (Figure 2). The accuracy of the DEM was verified by ground truth field survey data collected by using GPS with an accuracy of ± 3 meter error.

In order to validate the quality of measured soil texture data set, it was believed helpful to test the correlation between texture and other soil attribute, bulk density. Hand auger and core sampler were used to collect soil samples. At every sample point, the geographic coordinates, slope and elevation records were taken using GPS. The data were collected in January 2014, one month after crop harvesting. Samples were taken from a total of 36 sample locations. For texture analysis, a composite of 36 samples were collected from 0 to 30 cm depth. Each composite was made up from three separate samples taken from the same sample location, whereas two to three separate core samples (a total of 90 core samples across all sites) were taken for bulk density analysis.

### 2.3. Data Analysis

The soil samples were analyzed in the laboratory using the hydrometer method [28]. Bulk density (t/m^3^) was calculated as the dry weight of soil divided by its volume which includes the volume of soil particles and the volume of pores among soil particles.

The raw data set generated from laboratory analysis was subjected to Predictive Analytics Software (PASW) Statistics, using SPSS V. 20 (IBM Corp., Armonk, NY, USA). For data validation purpose, the value of soil texture and bulk density were subjected to Pearson correlation. In order to assess the appropriateness of the data set to the regression models, it was subjected to normality and homogeneity test of variance (error term) assumption tests using Kolmogorov-Smirnov and/or Shapiro-Wilk. In both cases, if the value of *p* was greater than 0.05, it was assumed that the data was normally distributed [29].

With one way analysis of variance (ANOVA), the extent of statistical variation in soil texture (clay and sand) among various terrain (slope and elevation classes separately) were compared. That is, group means differences for clay and sand were analyzed among the slope classes at a time and among the elevation classes at a time separately. Linear regression was employed to analyze and compare the strength of the relationship between terrain variables (slope and elevation) and soil texture. The standardized coefficient values were used to detect the degree of influence of slope and elevation on sand content. The tests were made at a confidence interval of 95%. The regression methods and their applications were explained by Gujarati et al. [29] and PASW Statistics, SPSS version 20.

## 3. Results and Discussion

### 3.1. Tests of Data Normality

Kolmogorov-Smirnov and/or Shapiro-Wilk attested the presence of normality, linearity (Table 2) of clay, silt and sand across elevation classes. The null hypothesis is that if there was no significant difference among groups variance, it will be rejected by the Test of Equality of Error Variances. The *p*-value for clay, silt, and sand across most of the elevation classes was 0.2. The test result assured the quality of the soil data set to proceed into ANOVA and linear regression analysis. Furthermore, Person correlation was run between soil texture (sand %) and bulk density (t/m^3^) to validate the output of the laboratory analysis. The dependence test was significant (*p* < 0.001) and the Pearson correlation value was 0.78.

The result of correlation analysis made between soil texture and bulk density attested the quality of soil sample data set. The finding was supported by the study of [15,30,31]. That is, sandy soil has less number of pore spaces, while clayey soils have more pore space due to the huge number of tiny pore spaces.

### 3.2. The Association between Soil Texture and Terrain

The statistical output showed an association between spatial variability of soil texture (clay and silt) among slope and agro-ecological zones (elevation) groups (Table 3). The extent of association was statistically significant (*p* < 0.05). Unlike what was observed in the case of elevation classes, the distribution of silt was not correlated to slope classes. In normal circumstances, unlike clay and sand particles, the size of silt particles is not expected to correlate with terrain attributes.

Like the one-way-ANOVA, the regression output indicated the presence of a significant association (*p* < 0.05) between soil texture (clay, silt and sand) and both terrain attributes. The standardized coefficient values of clay, silt, and sand attests that variation in texture was more pronounced among elevation values (Table 4, Table 5 and Table 6). In the estimation of clay particles, the standardized coefficients (Beta) value of slope and elevation were −0.499 and −0.767, respectively. Similarly, in the case of sand, the value of Beta for slope and elevation were 0.485 and 0.812 respectively.

The result of ANOVA and linear regression indicated the influence of terrain attributes over variation of physical soil properties among farmland plots. Referring to the USDA’s [17] soil texture triangle, heavy clay, clay and clay loam are identified as the major texture classes in the lower, middle and upper parts of the watershed respectively. As indicated by Lark et al. [32], the terrain attributes were significantly linearly related to physical properties of the soil. Similarly, in the finding of [23], the soil texture varied from sandy loam to clay in the surface horizons down the slope. This could be associated with the removal of fine soil particles from steeper slope and their deposition at lower slope positions. The loose aggregate nature of the upslope soils has enhanced easy movement of the fine soil particles downslope along with the rain water movement [23]. The finding of this study indicated that the catenary soil development in the watershed was well established. Most of the site is covered with clayey soil has poor circulation of water, nutrient and air in it. Tromp-van Meerveld et al. [16] and [17] were also stated that soil texture varied spatially with terrain attributes. As indicated by Cresswell et al. [30], such soil texture class is characterized by low available water holding capacity for reasons associated with compaction.

The result of linear regression of this study showed the presence of higher influence of the agro-climatic classes on the spatial distribution of soil texture than that of the slope classes. The effect of slope value variation on texture was more pronounced among the agro-ecological zones of the watershed than within an agro-ecological zone. The result of both statistical analyses implied that the most recent and dominant geomorphic process in the watershed was fluvial. The result implies the presence of more noticeable effect of agro-climatic zone variation (i.e., an interactive effect of major factors like microclimate and biota) on the spatial distribution of soil texture than that of the effect of slope class variation.

Moore et al. [33] explained the presence of differences in transport sediment particle size, whereby transport of course size particles was lower than that of fine particles. The studies made by [19,33] explained that clay content increases with decreasing slope and downstream flow length and with an increasing upslope flow length. This study had discovered a positive correlation between elevation and sand particles. 

Inconsistent to the finding of this study, areas with higher elevation presented higher values of clay content [22]. On the other hand, Dessalegn et al. [23] showed that the loose aggregate nature of the upslope soils has enhanced easy movement of the fine soil particles downslope along with the rain water movement. As reported by Kokulan et al. [34], elevation, relative slope position and texture at the surface were highly correlated. That is, increasing sand content and decreasing clay content with elevation was observed. Sand content was higher in the upslope positions and lower relative to silt and clay in the downslope areas, Tesfahunegn et al. [35] also presented that the highest values of fine soil particles were measured in the aggrading sites of the reservoir followed by the valley landform (south direction) and in the forested and afforested land systems of the catchment. Unfortunately, no previous study compared the influence of slope classes and elevation classes yet.

Taking into account the nature of texture, more clayey soil in the downstream part of the study site is expected to drain more water which permits longer growing periods to plants [15,33]. The upstream observed with relatively higher sand particles would have higher infiltration and less water holding capacity. This could happen due to the presence of soil with larger pores in sandy particles. The finding of this research happens in a situation where the topography is rugged (Figure 2) and the livelihood of people depends on subsistence food crop production.

Since the study watershed represents the agro-ecological feature of the northwestern highlands of the country, the statistical finding of the study could be useful information for the future related studies to be conducted in the larger tropical mountainous region. The observed result implies the presence of cultivated land use in steep slopes and siltation in the downstream. Unless corrective measures are taken in land use practices, in the long run, the existing situation could affect adversely the sustainability of crop land use and irrigation dam that may be constructed in the site.

## 4. Conclusions

Heavy clay, clay and clay loam are the major texture classes in the farmlands of the watershed. The spatial variability of soil texture has an association to both slope and elevation classes. Higher degree of soil texture variation was experienced among the agro-climatic zones (elevation) than slope classes. As we go down from the higher to lower elevation, the soil texture becomes more clayey. Lower slope areas show more clayey texture. The result implied the presence of high runoff and siltation in the upstream and downstream parts of the watershed. Applications of organic matter in the farmlands need to be a priority so as to improve the water infiltration capacity of the clayey soil. Taking into account this study conducted at a watershed level, it is very hard to generalize about the predictive power of the indicated terrain attributes on the spatial distribution of soil texture across the larger study area where the study watershed is situated. Hence, to generate a regional statistical model, further studies need to be conducted in the adjacent and diverse watersheds of the study site in Northwestern highlands of Ethiopia.

## Figures and Tables

**Table 1:** The local agro-ecological zones in Ethiopia and their agronomic features.

Agro-Ecological Zone	Elevation (m)	Annual Total Rainfall (mm)	Major Crops	Length of Growing Period (Day)
Arid lowland (Bereha)	<500	<900	sorghum, *nug ^1^* (Guizotia Abyssinica), *Dagusa ^2^* (Eleusine Coracana), groundnut	<120
Dry to Moist Lowland (Kolla)	500–1500	900–1400	sorghum, *nug*, groundnut	<120; 120–240
Dry to Wet Cool Highland (Weyna-Dega)	1500–2300	>900; 900–1400	*teff ^3^* (Eragrostis), maize, sorghu, *nug*, wheat, Dagusa, *inset ^4^* (*ventricosum)*, barley	<120; 120–240
Moist to Wet Cold Highland (Dega)	2300–2700	900–1400; >1400	barley, wheat & pulse	120–240; >240
Moist to Wet Alphine (Low-Wurch)	3200–3700	900–1400; >1400	barley	120–240; >240
High Alphine (High-Wurch)	>3700	>1400	none	>240

*^1^ Nug* is an annual herb grown for edible oil; *^2^ Dagusa* is named as finger millet in India; *^3^ Teff* is a traditional Ethiopian cereal), which is endemic to Ethiopia and Eritrea (although it is also grown in the U.S.A. today), and occupies about 20% of the cultivated land in Ethiopia [24]; *^4^ Inset* or false banana is a further endemic agricultural plant grown in higher-rainfall regions of the country in the Weyna Dega Belt.

**Table 2:** Test of normality for texture data sets across elevation classes.

Elevation		Kolmogorov-Smirnov ^a^			Shapiro-Wilk		
		Statistic	df	*p* Value	Statistic	df	*p* Value
Clay	Moist-Cool	0.158	14	0.200 *	0.965	14	0.797
	Cold	0.111	12	0.200 *	0.974	12	0.951
	Moist-Cold	0.432	6	0.001	0.687	6	0.005
	Sub-Alphine	0.306	4	-	0.768	4	0.056
Sand	Moist-Cool	0.150	14	0.200 *	0.949	14	0.540
	Cold	0.234	12	0.068	0.907	12	0.194
	Moist-Cold	0.216	6	0.200 *	0.874	6	0.245
	Sub-Alphine	0.283	4	-	0.863	4	0.272
Silt	Moist-Cool	0.234	14	0.037	0.885	14	0.068
	Cold	0.117	12	0.200 *	0.961	12	0.796
	Moist-Cold	0.178	6	0.200 *	0.979	6	0.946
	Sub-Alpine	0.304	4	-	0.811	4	0.123

* This is a lower bound of the true significance (*p* < 0.05); ^a^ Lilliefors Significance Correction.

**Table 3:** Test of ANOVA for texture data sets across slope and elevation groups.

Groups		Df	Slope	df	Elevation
			*p* Value		*p* Value
Clay %	Between Groups	5	0.023	3	0.00
	Within Groups	30		32	
	Total	35		35	
Silt %	Between Groups	5	0.158	3	0.27
	Within Groups	30		32	
	Total	35		35	
Sand %	Between Groups	5	0.047	3	0.00
	Within Groups	30		32	
	Total	35		35	

Dependent variable: clay, silt and sand.

**Table 4:** Test of linear regression for texture (clay %) and terrain attributes.

Model		Unstandardized Coefficients		Standardized Coefficients	*t*	*p* Value
		B	Std. Error	Beta		
1	(Constant)	68.519	4.571		14.990	0.000
	Slope	−4.413	1.315	−0.499	−3.356	0.002
2	(Constant)	76.689	3.496		21.939	0.000
	Elevation (m)	−10.889	1.563	−0.767	−6.966	0.000

Dependent variable: clay.

**Table 5:** Test of linear regression for texture (silt %) and terrain attributes.

Model		Unstandardized Coefficients		Standardized Coefficients	*t*	*p* Value
		B	Std. Error	Beta		
1	(Constant)	16.449	2.674		6.152	0.000
	Slope (%)	1.553	0.769	0.327	2.020	0.051
2	(Constant)	14.641	2.634		5.559	0.000
	Elevation (m)	3.299	1.178	0.433	2.801	0.008

Dependent variable: silt.

**Table 6:** Test of linear regression for texture (sand %) and terrain attributes.

Model		Unstandardized Coefficients		Standardized Coefficients	*t*	*p* Value
		B	Std. Error	Beta		
1	(Constant)	15.037	3.056		4.920	0.000
	Slope (%)	2.843	0.879	0.485	3.233	0.003
2	(Constant)	8.525	2.107		4.046	0.000
	Elevation (m)	7.638	0.942	0.812	8.107	0.000

Dependent variable: sand.
